# Predicting Cesarean Section and Uterine Rupture among Women Attempting Vaginal Birth after Prior Cesarean Section

**DOI:** 10.1371/journal.pmed.0020252

**Published:** 2005-09-13

**Authors:** Gordon C. S Smith, Ian R White, Jill P Pell, Richard Dobbie

**Affiliations:** **1**Department of Obstetrics and Gynaecology, Cambridge University, Cambridge, United Kingdom,; **2**Medical Research Council Biostatistics Unit, Institute of Public Health, Cambridge, United Kingdom,; **3**Department of Public Health, Greater Glasgow NHS Board, Glasgow, United Kingdom,; **4**Information and Statistics Division, Common Services Agency, Edinburgh, United Kingdom; Imperial College LondonUnited Kingdom

## Abstract

**Background:**

There is currently no validated method for antepartum prediction of the risk of failed vaginal birth after cesarean section and no information on the relationship between the risk of emergency cesarean delivery and the risk of uterine rupture.

**Methods and Findings:**

We linked a national maternity hospital discharge database and a national registry of perinatal deaths. We studied 23,286 women with one prior cesarean delivery who attempted vaginal birth at or after 40-wk gestation. The population was randomly split into model development and validation groups. The factors associated with emergency cesarean section were maternal age (adjusted odds ratio [OR] = 1.22 per 5-y increase, 95% confidence interval [CI]: 1.16 to 1.28), maternal height (adjusted OR = 0.75 per 5-cm increase, 95% CI: 0.73 to 0.78), male fetus (adjusted OR = 1.18, 95% CI: 1.08 to 1.29), no previous vaginal birth (adjusted OR = 5.08, 95% CI: 4.52 to 5.72), prostaglandin induction of labor (adjusted OR = 1.42, 95% CI: 1.26 to 1.60), and birth at 41-wk (adjusted OR = 1.30, 95% CI: 1.18 to 1.42) or 42-wk (adjusted OR = 1.38, 95% CI: 1.17 to 1.62) gestation compared with 40-wk. In the validation group, 36% of the women had a low predicted risk of caesarean section (<20%) and 16.5% of women had a high predicted risk (>40%); 10.9% and 47.7% of these women, respectively, actually had deliveries by caesarean section. The predicted risk of caesarean section was also associated with the risk of all uterine rupture (OR for a 5% increase in predicted risk = 1.22, 95% CI: 1.14 to 1.31) and uterine rupture associated with perinatal death (OR for a 5% increase in predicted risk = 1.32, 95% CI: 1.02 to 1.73). The observed incidence of uterine rupture was 2.0 per 1,000 among women at low risk of cesarean section and 9.1 per 1,000 among those at high risk (relative risk = 4.5, 95% CI: 2.6 to 8.1). We present the model in a simple-to-use format.

**Conclusions:**

We present, to our knowledge, the first validated model for antepartum prediction of the risk of failed vaginal birth after prior cesarean section. Women at increased risk of emergency caesarean section are also at increased risk of uterine rupture, including catastrophic rupture leading to perinatal death.

## Abstract 2

By studying a large group of Scottish women giving birth, Gordon Smith and colleagues developed and validated a tool to assess the risk of failed vaginal birth after prior caesarean section.

## Introduction

Encouraging women with a prior cesarean delivery to attempt vaginal birth in subsequent pregnancies is a strategy that has been employed to address rising rates of cesarean delivery. However, a series of retrospective studies published in the last five to ten years have indicated an increased risk of serious adverse outcomes among women who attempted vaginal birth compared with those who had a planned repeat cesarean delivery [1–3]. A recent large-scale prospective study has shown that among women with a prior cesarean delivery, the rates of maternal complications are highest among women who attempt vaginal birth and fail (14.1%), intermediate among women who have a planned cesarean delivery (3.6%), and lowest among women who attempt vaginal birth and succeed (2.4%) [4]. Therefore, the balance of risks and benefits of trial of labor versus planned repeat cesarean section itself depends on the risk of emergency cesarean section should labor be attempted.

Many studies have addressed methods for identifying women at low and high risk of failure of an attempted vaginal birth after a prior cesarean. A recent systematic review reported that only two of the six available tools had been validated [5]. Both of these incorporated data that would only be available when a women presented in labor, such as the results of electronic fetal monitoring and cervical dilatation on admission [6,7]. Currently, therefore, there is no validated antepartum tool to predict the risk of a failed attempt at vaginal birth among women with a prior cesarean delivery. Moreover, there are no data on whether women at increased risk of cesarean section are also at increased risk of uterine rupture. We sought to develop a simple, validated model to predict the risk of emergency cesarean section among women attempting vaginal birth and to determine whether women at increased risk of cesarean section were also at increased risk of uterine rupture, including catastrophic rupture leading to death of the infant.

## Methods

### Population

The Scottish Morbidity Record (SMR2) collects information on clinical and demographic characteristics and outcomes for all patients discharged from Scottish maternity hospitals. The register is subjected to regular quality assurance checks and has been more than 99% complete since the late 1970s [8]. A quality assurance analysis compared 1,414 records in 1996–1997 with the clinical notes. This analysis demonstrated that the register was free from significant errors in more than 98% of records in all the specific fields used in the present analysis, with the exception of postcode (94.0%), height (96.2%), estimated gestation (94.4%), and method of induction of labor (93.6%). The previous cesarean section field was 99.7% accurate. International classification of disease (ICD) diagnostic codes were found to be 80%–90% accurate for the first four diagnoses and 70%–80% accurate for the remainder [9]. SMR2 records were linked to records from the Scottish Stillbirth and Neonatal Death Enquiry. This national register has routinely classified all perinatal deaths in Scotland since 1983. It is virtually 100% complete and has been described in detail elsewhere [10]. The predictors of cesarean section employed in the current study were those that were recorded in the SMR2 and which had been identified in previous studies as possible risk factors for emergency cesarean delivery.

### Study Group

The population was drawn from all term singleton births to women with one prior cesarean section in Scotland between 1985 and 2001, inclusive. The exclusion criteria for the study group were preterm birth, perinatal deaths due to congenital anomaly, antepartum stillbirth due to any cause, deliveries by planned cesarean section, and women documented as being primigravid despite also being documented as having had a prior cesarean delivery. The primary analysis was confined to women who delivered at or after 40-wk gestation.

### Definitions

Emergency cesarean section was defined as any non-planned cesarean delivery. Maternal age was defined as the age of the mother at the time of birth. Maternal height was measured in centimeters, and the value used was that documented in each woman's clinical record. Gestational age at birth was defined as completed wk of gestation on the basis of the estimated date of delivery in each woman's clinical record. Gestational age has been confirmed by ultrasound in the first half of pregnancy in more than 95% of women in the United Kingdom since the early 1990s [11]. Hospital throughput was defined as the total number of births recorded in the SMR2 database for the given hospital over the given year and was categorized into above or below the national median (3,000 births).^9^

Perinatal deaths were classified on the basis of data from the Scottish Stillbirth and Neonatal Death Enquiry [10]. Death caused by congenital anomaly was defined as any structural or genetic defect incompatible with life or potentially treatable but causing death. The registry subclassifies stillbirths into antepartum (deaths before the onset of labor) and intrapartum (deaths during labor). A death was taken to be due to intrapartum uterine rupture if it was an intrapartum stillbirth or neonatal death, when the cause of death was documented as intrapartum anoxia, when the obstetric cause of death was coded as “mechanical” under the modified Wigglesworth classification [12], and when the ICD9 code for intrapartum uterine rupture (665.1) was listed as a specific diagnosis. Intrapartum uterine ruptures not resulting in perinatal death were identified using ICD9 and ICD10 diagnostic codes 665.1 and O711, respectively, from the diagnostic fields in the SMR2 record related to hospital discharge following delivery.

### Statistical Analyses

Continuous variables were summarized by the median and interquartile range (IQR), and comparisons between groups were performed using the Mann-Whitney *U* test. Univariate comparisons of categorical data were performed using Fisher's exact test. The *p* values for all hypothesis tests were two-sided. The risk of adverse outcomes was modeled using multivariate logistic regression [11]. First order interactions were assessed using the likelihood ratio test and significance assumed at *p* < 0.05 after correction for the number of comparisons using the Bonferroni method. The goodness of fit of logistic regression models was assessed using the Hosmer and Lemeshow test. Assessment of linearity of age and height in logistic models was performed using fractional polynomials. Cases with extreme values of age or height (≤0.1 percentile and ≥99.9 percentile) were excluded. Out-of-sample validation of the model was performed by dividing the cohort into model development and model validation groups. Models were constructed for the development group and the predicted numbers of cesarean sections were related to the observed number of events in the validation group when categorized into deciles of predicted probability. Selection of model development and validation groups was initially random and the process was then repeated selecting the groups on specific characteristics (hospital throughput, deprivation category and year of delivery). Random allocation into two groups was performed using a pseudo-random number. The predictive ability of models was assessed by the area under the receiver operating characteristic (ROC) curve, and curves were compared using the algorithm described by De Long et al [12]. The final logistic regression model fitted to the entire cohort was expressed as adjusted log likelihood ratios (ALLRs) using a modification of our recently described method [13] (see Supporting Information for details). Logistic regression analysis of the risk of perinatal death was performed using exact logistic regression due to the rarity of the event. All statistical analyses were performed using the Stata software package version 8.2 (Stata Corporation, College Station, Texas, United States), except exact logistic regression which was performed using LogExact version 5.0.1 (Cytel Software Corporation, Cambridge, Massachusetts, United States).

## Results

Between 1985 and 2001, 68,380 women delivered who had one prior cesarean delivery. We excluded 150 (0.2%) births outside the range 24 to 43 wk, 4,700 (6.9%) preterm births, 366 (0.5%) antepartum stillbirths, 21,677 (31.7%) women delivered by planned cesarean section, 124 (0.2%) women whose infant was a perinatal death attributed to a congenital abnormality, and 76 (0.1%) women documented as being primigravid. A total of 25,836 (37.8%) women had one or more of these exclusions, leaving 42,544 (62.2%) women. Among the 25,964 (61.0%) women who delivered at or after 40-wk gestation, 2,585 (10.0%) had a missing value for height, one (<0.1%) had a missing value for age, 51 (0.2%) had an extreme value of height, and 41 (0.2%) had an extreme value of maternal age, leaving 23,286 women eligible for study. These women were randomly allocated to a model development or model validation group, and the demographics and basic outcome data for the cohort were tabulated (Table 1). Women who had previously had a vaginal birth were older than those with no previous vaginal birth (median IQR: 30 [27–34] versus 29 [25–32], respectively, *p* < 0.001).

**Table 1:**
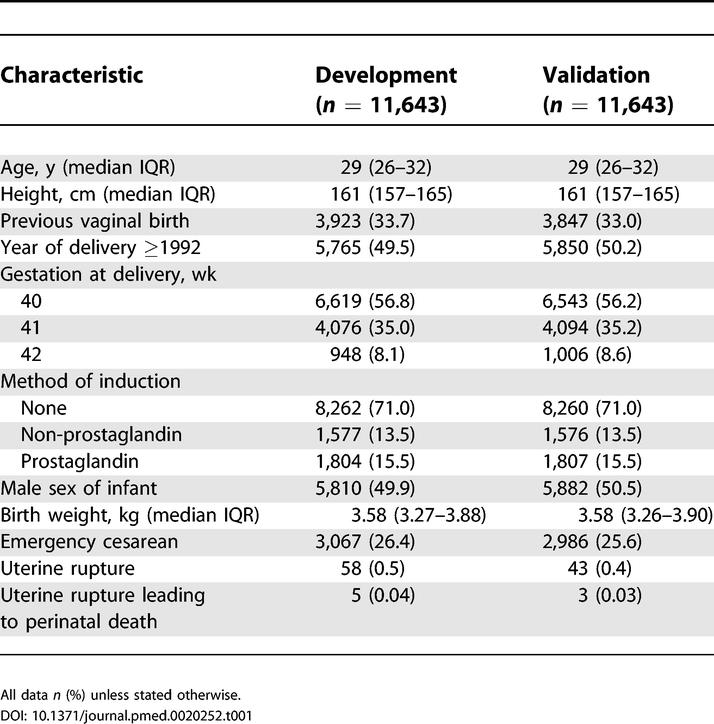
Characteristics of Population by Allocation to Development or Validation Group

In univariate and multivariate analysis in the model development group, all factors were significantly associated with the risk of emergency cesarean section except induction of labor using a means other than prostaglandin (Table 2). The area under the ROC curve in the development group was 0.706, which was significantly greater than for any of the individual predictors (all *p* < 0.001). There were no statistically significant first order interactions between the predictors. When the model was applied to the validation group, the area under the ROC curve was 0.708 (Table 3). The observed proportion of emergency cesarean deliveries and the proportion predicted by the multivariate model derived from the model development group were similar (Figure 1A). In the validation group, 36% of the women had a low predicted risk of caesarean section (<20%) and 16.5% of women had a high predicted risk (>40%); 10.9% and 47.7% of these women, respectively, actually had deliveries by caesarean section.

**Figure 1:**
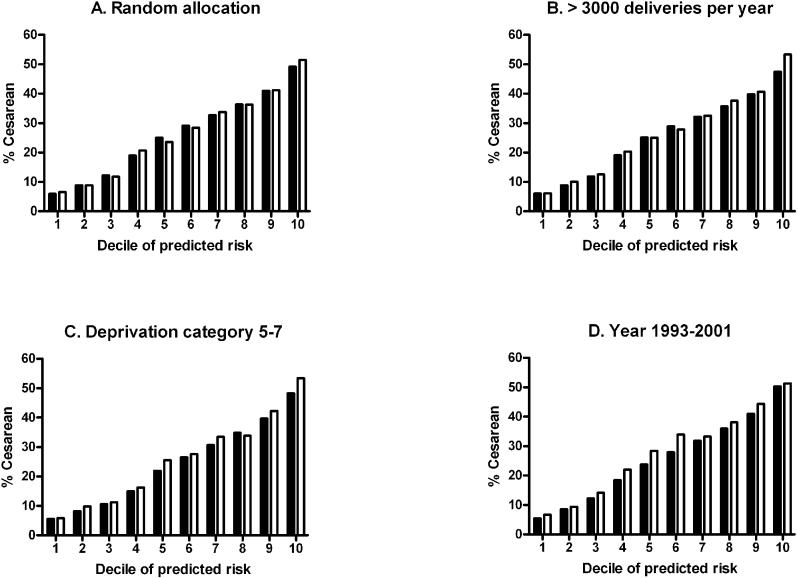
Observed and Expected Proportion of Cesarean Deliveries in the Model Validation Group by Decile of Predicted Probability The white bars indicate the observed proportion and the black bars indicate the expected proportion of cesarean deliveries, based on estimates derived from logistic regression model fitted to the development group. Different graphs represent different procedures for selecting development and validation groups: (A) random selection, (B) selected on hospital throughput, (C) selected on deprivation category (Carstairs score), and (D) selected on year of delivery. Area under the ROC curve for each model is listed in Table 3.

**Table 2:**
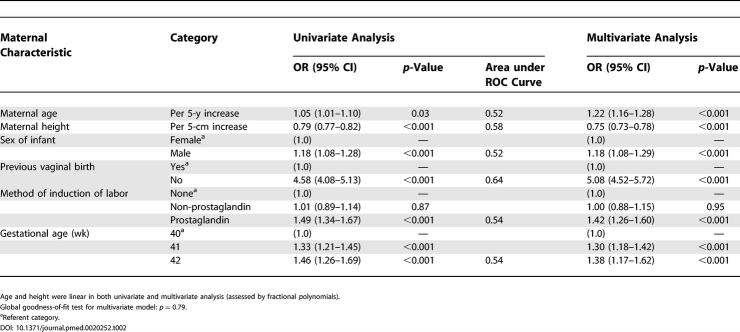
Univariate and Multivariate Analysis of Predictors of Emergency Cesarean Section in the Model Development Group (*n* = 11,643)

**Table 3:**
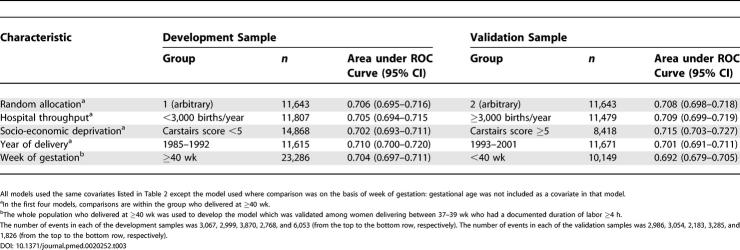
Assessment of the Modeling Approach in Development and Validation Samples

The process of model development and validation was then repeated with nonrandom selection of the development and validation samples. Three nonrandom procedures for selection were evaluated, namely, hospital throughput (<3,000 births per year and ≥3,000 births per year), deprivation category (Carstairs category <5 and Carstairs category ≥5), and year of birth (1985–1992 and 1993–2001). The area under the ROC curve was similar when the development and validation samples were compared, (Table 3) and when the data were plotted according to the predicted risk, the expected and observed number of cesarean deliveries were similar in the validation samples (Figure 1B–1D).

A logistic regression model was then fitted for the whole cohort. The area under the ROC curve was 0.707 and the global goodness-of-fit test showed no evidence of poor fit (*p* = 0.95). The output was converted to ALLRs (Table 4) using a modification of our previously described method [13]. The calculation of a summary ALLR for a series of maternal characteristics is illustrated in the box. The summary ALLR could also be used in combination with a published nomogram to generate a predicted probability [14]. Assuming a prior probability of emergency cesarean delivery of 26%, a summary ALLR of 0.71 or less was associated with a less than 20% chance of emergency cesarean section, and a summary ALLR of 1.91 or more was associated with a greater than 40% chance of emergency cesarean section.

**Table 4:**
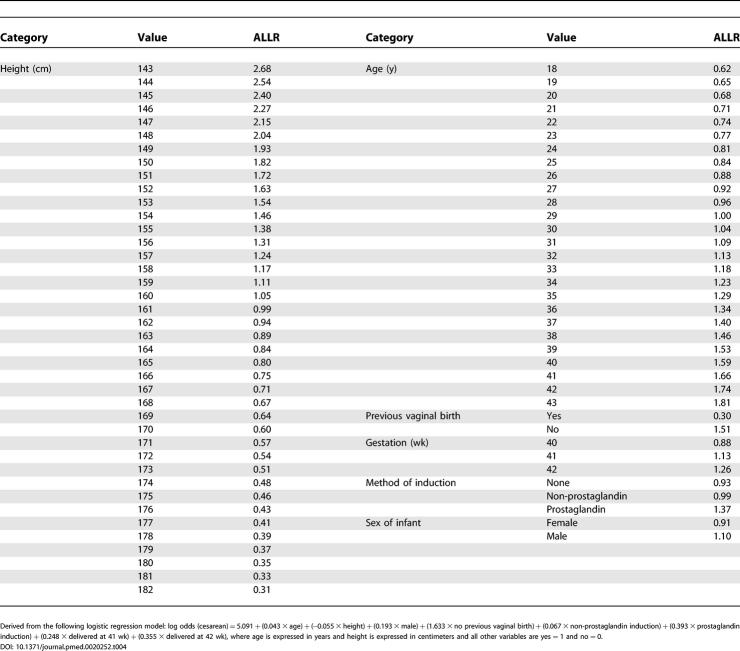
ALLRs for Maternal Characteristics and Fetal Sex Derived from Logistic Regression Model Fitted for the Whole Population

The probability of cesarean section was calculated for each woman in the cohort using the multivariate model. We then analyzed the risk of uterine rupture in relation to the predicted risk of emergency cesarean section. The predicted probability of cesarean section was also associated with the risk of all uterine rupture (Figure 2; odds ratio for a 5% increase in predicted risk = 1.22, 95% confidence interval [CI]: 1.14 to 1.31) and uterine rupture associated with perinatal death (odds ratio for a 5% increase in predicted risk = 1.32, 95% CI: 1.02 to 1.73). Among women with a predicted cesarean section risk of less than 20%, the incidence of uterine rupture was 2.0 (95% CI: 1.1 to 3.2) per 1,000, and among women with a cesarean section risk of greater than 40%, the incidence of uterine rupture was 9.1 (95% CI: 6.4 to 12.6) per 1,000, relative risk 4.5, (95% CI: 2.6 to 8.1).

**Figure 2:**
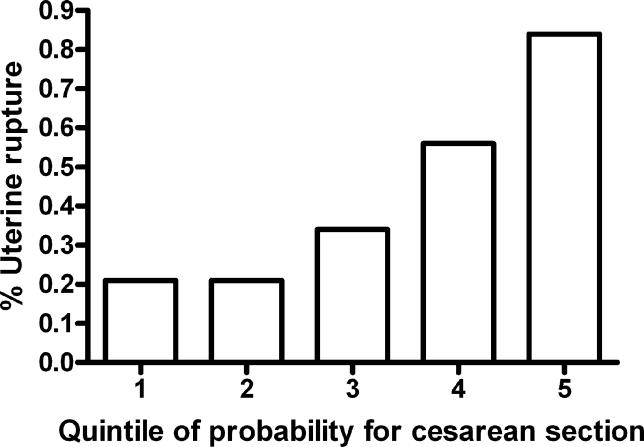
Proportion of Uterine Ruptures in Relation to the Quintile of Predicted Probability of Emergency Cesarean Delivery for the Whole Population *n* = 23,286; *p* < 0.001 (Chi square test for trend).

The population studied had excluded women who delivered at 37- to 39-wk gestation. A model (excluding week of gestation) was fitted for women delivering between 40- and 42-wk and was evaluated among women delivered at 37- to 39-wk gestation in whom the documented duration of labor was greater than or equal to 4 h but otherwise applying the same inclusion and exclusion criteria as the main study cohort. The area under the ROC curve was 0.692. Among the 10,147 eligible women who delivered at 37 to 39 wk, there were 1,826 cesarean deliveries (18.0% compared with 26.0% in the rest of the population), giving a pretest odds of 0.22. When the probability of cesarean section was estimated using a prior odds of 0.22 and the ALLRs listed in Table 4 (excluding week of gestation), the observed and expected number of cesarean deliveries were similar (Figure 3).

**Figure 3:**
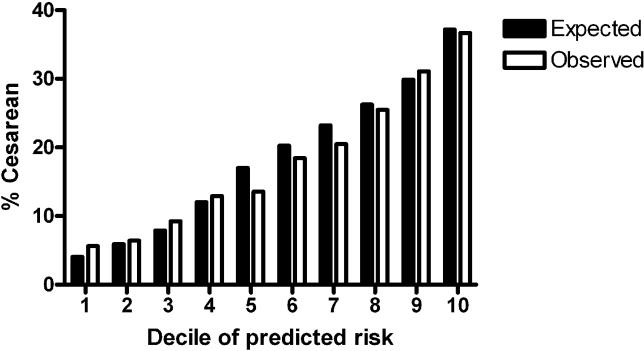
Use of ALLRs to Predict Probability of Cesarean Section among Women Delivered 37 to 39 wk with a Documented Duration of Labor of Greater than or Equal to 4 h Probability estimated using likelihood ratios in Table 4 (excluding gestational age) and the prior odds of 0.22 (equivalent to background risk of cesarean section in this group).

## Discussion

Women who have had a prior cesarean delivery need to choose whether to have a planned repeat cesarean section or to attempt vaginal birth in subsequent pregnancies. The risk of maternal morbidity depends on whether the attempt at vaginal birth is successful [1,4]. An informed discussion of this decision requires an assessment of the risk of emergency cesarean section. However, there is, at present, no validated method that allows antepartum assessment of the risks of emergency cesarean section [5], and counseling of women is, at best, semiquantitative. In the present study we provide a validated model that classifies over half this population as being at low or high risk of emergency cesarean section, on the basis of thresholds suggested by a previous systematic review [5]. When the model was validated, 36% of women had a predicted risk of cesarean section of less than 20%, and their overall cesarean section rate was 10.9%. Conversely, 16.5% of women had a predicted risk of cesarean section of greater than 40%, and their overall cesarean section rate was 47.7%.

One of the other principal concerns among women who have had a prior cesarean section is the risk of intrapartum uterine rupture. Uterine rupture is associated with an increased risk of severe maternal complications, such as hysterectomy and hemorrhage [1,4] and with an increased risk of severe effects on the infant, including hypoxic ischemic encephalopathy[4] and perinatal death [9]. Even if a woman had a low risk of emergency cesarean section, she may choose to have a planned repeat cesarean section due to concerns about the possibility of uterine rupture. However, we found that women who were at low risk of emergency cesarean section were also at low risk of uterine rupture, including catastrophic rupture leading to perinatal death. Among women with a predicted cesarean section risk of less than 20%, the incidence of uterine rupture was 2.0 per 1,000, whereas among women with a cesarean section risk of greater than 40%, the incidence of uterine rupture was 9.1 per 1,000. This is the first study, to our knowledge, to demonstrate a direct association between the risk of a failed attempt at vaginal birth and the risk of uterine rupture. This cannot be explained by ascertainment bias because the association was still apparent when the analysis was confined to catastrophic uterine rupture leading to death of the infant, which would be ascertained irrespective of the mode of delivery.

In order for this model to be clinically useful, it needs to be presented in a way that practicing clinicians can understand and apply. To this end, we have employed a method for converting the logistic regression model into ALLRs. These can be used like conventional likelihood ratios and an example is given in the box. The prior odds are multiplied by the appropriate ALLRs to give the posterior odds from which the probability of cesarean section can be derived. Because this method is very similar to that used for Down syndrome screening, we feel that it is likely to be generally understood by practicing clinicians. A previous method has been described in detail to convert logistic regression models into a Bayesian format [15]. This method provides identical results to our method for simple models. However, for models with categorical variables containing three or more groups or in which the scaling of a continuous variable changes between the univariate and multivariate analysis, the previously described method does not generate identical estimates of probability. Our method always generates estimates of probability that are identical to the logistic regression model. It can be thought of, therefore, as a simple format for the presentation of logistic regression models.

The present study has a number of strengths over previous studies. First, we had a population of over 23,000 women. The largest previous study included approximately 5,000 women [6]. Second, we were able to ascertain uterine rupture in our population, including uterine rupture leading to perinatal death. Studies using registry-based data have the profound weakness that uterine rupture may be inconsistently defined and include cases of avascular wound dehiscence detected at the time of cesarean delivery [2]. This study design could lead to ascertainment bias because women having cesarean delivery would be more likely to have avascular wound dehiscence identified. However, our data sources allowed us to identify uterine ruptures that led to death of the infant. Third, because of the large numbers, we could confine the analysis to women delivered at or after 40-wk gestation. Large-scale registries lack details such as whether an attempt at vaginal birth was planned. Planned cesarean sections are typically performed at 38–39 wk in the United Kingdom [16]. By confining the analysis to births at or after 40 wk, we could exclude women who were not truly attempting vaginal birth. Fourth, the risk of cesarean section could be estimated using information available in the antepartum period. Counseling of women regarding vaginal birth frequently involves the distinction between attempting vaginal birth if the onset of labor is spontaneous but not attempting it if labor needs to be induced, particularly if prostaglandins are used to ripen the cervix [17]. For this reason, we included week of delivery and method of induction of labor in the model, and, therefore, the current model can inform such decisions. Excluding gestation and mode of induction had very little practical effect on the predictive ability of the model (data not shown). Fifth, we analyzed continuous variables continuously rather than categorizing them, which increases statistical power. This analysis may explain why we observed a positive association between maternal age and risk of cesarean section, whereas some other studies have not [7]. Interestingly, the association with age became much stronger in multivariate analysis. This result may reflect negative confounding by previous vaginal birth. This factor was strongly protective against cesarean delivery, and these women were significantly older than other women.

The present study has some weaknesses. First, the data were obtained from Scotland and there may be concerns in applying this model to other populations. However, we assessed the robustness of the predictors employed by selecting records for the development and validation groups on the basis of factors that might reflect variation in other populations. We found the model was similarly predictive in and out of sample when these categorizations were performed by hospital throughput, socio-economic deprivation category, and year of birth. This finding suggests that the maternal and obstetric characteristics used in the model are likely to be robust even when applied to populations with different obstetric practices. Second, we lacked data on other factors that might be predictive of the risk of emergency cesarean delivery, such as body mass index, the indication for the previous cesarean section, and whether a previous vaginal birth preceded or followed the previous cesarean section. Nevertheless, we report the first validated model to give useful discrimination of risk to greater than 50% of the population [5].

A further potential weakness with the model is that it was derived from women delivering at or after 40-wk gestation. As discussed above, we confined the primary analysis to this group in order to identify women who were truly attempting vaginal birth. Some women who were scheduled for planned cesarean section will have attended prior to this date in labor. Such women would be documented as an intrapartum emergency cesarean section but did not truly attempt vaginal delivery. However, we needed to assess the validity of the model for women who deliver at earlier week of gestation at term. Another means to identify women who were truly attempting vaginal birth is to confine analysis to those with a documented duration of labor of at least 4 h. We evaluated the model in women who were delivered between 37 and 39 wk who had labor for 4 h or longer. The discriminative power of the model was comparable, with an area under the ROC curve of 0.692. These women had a lower prior risk of cesarean section (18%) than women at or after 40-wk gestation (26%). When ALLRs were employed and the lower overall rate of cesarean delivery was accounted for by using the prior odds of 0.22, the observed and expected numbers of cesarean deliveries were similar (Figure 3). We conclude that the ALLR-based model is appropriate for births between 37–39 wk if lower prior odds of cesarean delivery are employed. Moreover, this analysis highlights one advantage of an ALLR-based approach, namely, that it is simple to adjust the estimate of probability for a lower or higher prior odds of the outcome.

In conclusion, we present a simple, validated model for clinical estimation of the risk of emergency cesarean section among women with a prior cesarean delivery attempting vaginal birth. Women at high risk of cesarean delivery are also at increased risk of uterine rupture, including catastrophic rupture leading to perinatal death.

## Supporting Information

The full logistic regression model for calculating ALLRs is



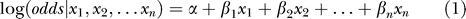



where *x*_1_, *x*_2_,…, *x_n_* are the predictor variables, β_1_, β_2_,…, β*_n_* are their regression coefficients, and α is the constant. Let the fitted values of α, β_1_, β_2_,…, β*_n_* be 
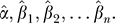
.

The log likelihood ratio for *x*_1_, for example, may be defined as the log odds of the outcome conditional on *x*_1_, *x*_2_,…, *x_n_* minus the log odds of the outcome conditional on *x*_2_,…, *x_n_*. The latter odds cannot in general be derived from equation 1 because the effects of the omitted *x*_1_ are partly picked up by *x*_2_,…, *x_n_*: the true likelihood ratio for *x*_1_ therefore depends on the values of *x*_2_,…, *x_n_*. We have created ALLRs that do not depend on the values of *x*_2_,…, *x_n_*.

To create the ALLRs, we force the coefficients in the second model to be the same as those estimated in the first model, but allowing a different intercept:



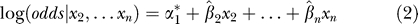



In this model, only the parameter 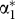 is to be estimated; the other parameters take their fitted values from equation 1. We can then calculate the ALLR for *x*_1_ as



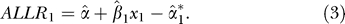



This procedure is repeated for each variable *x*_2_,…,*X_n_* to calculate *ALLR*_2_,…, *ALLR_n_*

A small correction factor must be added to the ALLRs in order to ensure that the sum of the overall log odds and all the ALLRs is exactly equal to the log odds computed from equation 1. The appropriate correction factor is *c_i_d,* where 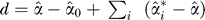, α̂ is the overall log odds, and Σ_*i*_ *c_i_* = 1. In this paper, *d = −*0.021 and all correction factors were smaller than 0.01 in magnitude. To ensure that values of each *ALLR_i_* straddle 1, *c_i_* is calculated as *m_i_*/(*m*_1_ +…+ *m_n_*) where *m_i_* is the sample minimum or maximum (depending on whether *d* is positive or negative) of *ALLR_i_*.

At the end of this procedure, the sum of the overall log odds and all the ALLRs exactly equals the log odds computed from equation 1. Our procedure is therefore nothing more than a restatement of the results of the logistic regression in an easily interpretable format.

**Patient Summary**

**Background**

The number of cesarean sections performed is increasing. Many women who have had a previous cesarean section want to try to have a vaginal birth in the next pregnancy, but they and their doctors may be worried about whether or not it is safe for them and the baby to attempt the vaginal birth.

**What Did the Researchers Do and Find?**

They looked at a large number of women in Scotland who had had one previous cesarean section and who were about to have another baby. Altogether they studied 23,286 women who had attempted to give birth vaginally between 1985 and 2001. They split the women into two groups; using one group, they developed a way of predicting the outcome (whether or not the women were going to need an emergency cesarean section) by looking at various risk factors including mother's age, height, sex of baby, gestation, and whether and how the birth was induced. Then, using the second group of women, they tested the model they had developed. They discovered that they could identify half of the women as being at high or low risk of needing emergency cesarean section, with the remainder being at intermediate risk. The things that increased risk were older maternal age, smaller height, male sex of baby, labor induced by prostaglandin, not having had a previous vaginal birth, and later birth. They also found that the risk of having a ruptured uterus went up as the risk of emergency cesarean section went up.

**What Do These Findings Mean?**

Obstetricians will be able to use the model developed to try to give women a more accurate estimate of whether they will need to have a cesarean section once they have had one in a previous pregnancy.

**Where Can I Get More Information Online?**

The following Web sites have relevant information.

MedlinePlus has a selection of pages with information for patients:

http://www.nlm.nih.gov/medlineplus/cesareansection.html

The United Kingdom's OMNI gateway has links to sites about cesarean section:

http://omni.ac.uk/browse/mesh/D002585.html

**Box 1.**

**Sample Calculation**

Background risk of cesarean section = 26%. Convert into odds if prior odds of cesarean section = 26/74 = 0.35

**Example**

A 37-y-old woman, 160 cm tall, with no previous vaginal birth, and with a male infant wishes to know probability of cesarean section if she requires induction of labor at 41 wk gestation using prostaglandin.

**Summary**

ALLR = 1.40 × 1.05 × 1.51 × 1.10 × 1.13 × 1.37 = 3.78. Posterior odds = 0.35 × 3.78 = 1.32. Chance of cesarean delivery = 1.32/(1 + 1.32) = 0.57 or 57%. (This is identical to the estimated risk using the logistic regression equation in the footnote of Table 4).

## Abbreviations

ALLR: adjusted log likelihood ratio
CI: confidence interval
ICD: International classification of disease
IQR: interquartile range
OR: odds ratio
ROC: receiver operating characteristic
SMR2: Scottish Morbidity Record
